# School and local authority characteristics associated with take-up of free school meals in Scottish secondary schools, 2014

**DOI:** 10.1080/21582041.2016.1223871

**Published:** 2016-09-30

**Authors:** Stephanie Chambers, Ruth Dundas, Ben Torsney

**Affiliations:** ^a^ MRC/CSO Social & Public Health Sciences, University of Glasgow, Glasgow, UK; ^b^ School of Mathematics and Statistics, University of Glasgow, Glasgow, UK

**Keywords:** free school meals, benefits take-up, inequalities, stigma, utility-maximisation, multilevel modelling

## Abstract

School meals are an important state-delivered mechanism for improving children’s diets. Scottish local authorities have a statutory duty to provide free school meals (FSM) to families meeting means-testing criteria. Inevitably take-up of FSM does not reach 100%. Explanations put forward to explain this include social stigma, as well as a more general dissatisfaction amongst pupils about lack of modern facilities and meal quality, and a preference to eat where friends are eating. This study investigated characteristics associated with take-up across Scottish secondary schools in 2013–2014 using multilevel modelling techniques. Results suggest that stigma, food quality and the ability to eat with friends are associated with greater take-up. Levels of school modernisation appeared less important, as did differences between more urban or rural areas. Future studies should focus on additional school-level variables to identify characteristics associated with take-up, with the aim of reducing the number of registered pupils not taking-up FSM.

## Introduction

UK local authorities have a statutory responsibility to provide meals at lunchtime in schools. In addition, children whose families are in receipt of certain benefits or tax credits, and/or have a low household income are entitled to receive their meal for free. Children aged 16–18, who are in receipt themselves of benefits, are eligible also. Universal provision of school meals for children in their first three years of primary school is in place in Scotland and England. Older children continue to pay for their meals when their family do not meet the eligibility criteria. Whether a means-tested or a universal system is in place, take-up of school meals at a national level will never reach 100%. There are inevitably families and children who choose different arrangements, whether they receive their meal for free or pay for it.

### History of school meal provision

School meal provision was introduced to meet the nutritional needs of poor children in the early twentieth century. Before this time, school food provision had been largely a charitable endeavour (Harris, 1995). State provision moved in line with a shift in the discourses surrounding school food from that of poverty alleviation and attainment, to a concern about undernutrition (Atkins, 2007).

Before the 1906 Education Act, many parents did not take up an FSM due to fears of being labelled ‘paupers’ (Harris, 1995). To tackle the fear of stigma, a system of school medical officer assessment was introduced to determine whether a child was malnourished, and if they would benefit from receiving an FSM. The system was greatly criticised, as school medical officers argued that children needed to reach a malnourished state before they were given a means to exit it (Hurt, 1985). Under pressure, the system was changed to one of financial, rather than nutritional, need for entitlement, and has remained so until the present day. Opposition to FSM subsided as the welfare state expanded in the post Second World War period, with food quality improving, and through the 1960s and 1970s, recommendations were made to increase the nutritional value of meals further. The 1980 Education Act removed the duty on local authorities to provide meals to all children, and required them to provide meals only for children who were eligible to receive them for free. In 1986, eligibility for FSM was restricted to families receiving Income Support.

### Nutritional concerns and school meals

In the early twenty-first century, the main debate surrounding school meals is the adequacy of the nutrition they provide. The changes to arrangements in the 1980s, including the introduction of compulsory competitive tendering, and the abolition of nutritional standards, saw a perceived decline in the quality and nutritional value of the food on offer (Scottish Government, n.d.). National surveys highlighted how far UK diets were from recommendations for key macro and micro-nutrients, and increases in overweight, obesity and chronic conditions indicated that widespread changes were needed (Hoare et al., 2002). Under pressure from high-profile campaigns to improve the nutritional value of school food, nutrient standards were introduced by the UK and devolved governments (Morgan & Sonnino, 2008). Cost–benefit analyses suggest that investment in improving the nutrient density of the foods on offer in schools can provide long-term economic gains through increased health and productivity (Nelson, 2013). Increasing take-up of school meals is perceived as especially important due to concerns over the nutritional value of packed lunches. Evidence suggests that packed lunches are less likely to contain healthy food, and more likely to contain high-energy foods and drinks compared with school lunches (Evans, Mandl, Christian, & Cade, 2016). In addition, for those children receiving FSM, a higher proportion of their energy and nutritional needs are met through a school lunch than for children not registered for FSM, suggesting that for children registered for FSM, school lunch is their main meal of the day (Stevens & Nelson, 2011).

### Utility-maximisation theory and non-take-up of social benefits

FSM are one of many benefits available to UK citizens, and whether these benefits are universal or targeted, take-up rarely reaches 100%. Moffit (1983) has identified stigma and/or disutility as explanations for lack of take-up of benefits. He distinguishes two types of stigma: fixed and variable. Fixed stigma relates to the potential shame felt by participation in a programme, whereas variable stigma varies as a function of the benefit received. Reduction of stigma has been a central component of Scottish school meals policy, with the principle enshrined in legislation. Local authorities must ensure that children eligible to receive FSM are not identified as being so. Many local authorities introduced cashless systems to ensure the protection of confidentiality, but these systems are not in place uniformly.

Kerr (1982) outlined a typology of thresholds that must be met before a benefit is taken up. After perceived need, basic knowledge and perceived eligibility (all of which have been established by the time a pupil is registered for FSM), the participant must perceive the utility of the benefit, and have a positive perception that the social outcomes will result in an overall benefit. The use of utility-maximisation theory, or a trade-off between barriers and facilitators, to explain non-take-up has been supported in a number of studies (Ritchie, 1988; Tempelman & Houkes-Hommes, 2015; Whelan, 2010). Interestingly, Tempelman and Houkes-Hommes (2015) found that it was not necessarily amongst those with the lowest incomes or greatest need that benefits take-up was highest. With reference to FSM, Macdiarmid et al. (2015) found that the likelihood of children buying food outside school at lunchtime, rather than in the dining hall, was greatest in areas of highest deprivation. However, research suggests that take-up of benefits is more likely the higher the monetary benefit gained (Tempelman & Houkes-Hommes, 2015). FSM is unlike other benefit take-up research, as parents have already met the administrative cost associated with completing the appropriate forms, declaring their receipt of benefits, tax credits and/or their low income. Children need to only attend school and receive their meal for free. Within a utility-maximisation framework, we must look instead at the other potential costs to pupils involved in taking-up FSM. These ‘costs’ are outlined below.

### Non-take-up of school meals

There has been only limited discussion as to why take-up of FSM does not reach 100%. Research has focused at the general level on why all pupils might wish to eat outside school, or to take a packed lunch option as an alternative. Nevertheless, this work provides insight into some of the transactional costs that pupils might face when they take-up their entitlement to an FSM.

Stigma is the single issue that relates directly to pupils registered for FSM. In many schools, traditional administrative arrangements provided pupils with tickets distinguishing them from classmates whose parents’ paid for their meals. With policy changes and greater awareness, such practices are less common, with many schools operating cashless systems. Nevertheless, where cash systems remain, it is often possible to identify which pupils are receiving their meal for free. In spite of fears around stigma, research suggests, however, that it is not a significant issue affecting pupils at either primary or secondary level (Sahota, Woodward, Molinari, & Pike, 2014). Sahota et al. (2014) found that where registration for FSM was high, the practice of taking-up the benefit was normalised, suggesting that not feeling singled out potentially impacts take-up decisions.

A more general concern affecting all school children is the dining hall environment. The issue of dining hall size is one of the most challenging for schools and local authorities to manage. At secondary level, it is often impossible for all pupils in the school to be seated at once, with staggered servings in place and limited time allocated to eat. In busy dining halls, pupils are less able to sit with friends and enjoy the valued social aspects of lunch (Wills et al., 2015; Wills, Danesi, Kapetanaki, 2015; Young, Gilligan, & Bainbridge, 2014). Serving areas can also be limited, resulting in larger queues, particularly off-putting to secondary school children (Sahota et al., 2014; Young et al., 2014). In addition, busy dining halls inevitably result in high noise levels, with the acoustics in high-ceilinged, multi-use dining spaces particularly problematic. Another issue raised is the institutional feel of many dining halls, which impacts on the important social value pupils attach to lunchtime (Burke, 2005). In Scotland, investment has been made in school buildings, although many local authorities are in the middle of rebuilding programmes, and are yet to realise the full benefits of more modern facilities.

The introduction of nutrient standards for school meals has been hailed as an important public health measure. In the period following their introduction, news reports detailed dramatic decreases in take-up as children turned away from the healthier meals on offer (BBC News, 2007). Public consciousness was particularly gripped at this time by celebrity chef Jamie Oliver’s campaign around improving the overall and nutritional quality of the meals offered within schools (Morgan & Sonnino, 2008). Although take-up did initially dip after the changes were introduced, it has recovered somewhat. Day-to-day, however, take-up is menu dependent, and children are more likely to eat on the days in which popular items are on offer. Issues of greatest concern around food for secondary pupils are lack of meal options, preferences for less healthy meals, small portion sizes, and the limitations of the FSM allowance for purchasing additional foods and drinks (Sahota et al., 2014; Wills, Kapetanaki, et al., 2015; Young et al., 2014).

When children do not wish to eat in the school dining hall, or to eat the foods provided there, they inevitably find other outlets in which to spend money, even when they are entitled to receive a free meal. It is estimated that there are an average of 35 food retailers within an 800 metre radius of secondary schools in Glasgow, Scotland’s largest city (Ellaway et al., 2012). Macdiarmid et al. (2015) surveyed secondary school pupils about why they purchase lunch from outlets around schools rather than eat in the school dining hall. The explanations provided included variety, value for money, and to purchase foods and drinks not available in schools. The most important reason cited was to spend time with friends, a finding also identified by Wills, Kapetanaki, et al. (2015). In comparison with children who never purchase food or drinks from outside their school at lunchtime, those who do consume a higher intake of added sugar as a percentage of food energy (Macdiarmid et al., 2015). With this in mind, some secondary schools prohibit first year pupils from leaving school premises at lunchtime (Scotcen, 2011). Pupils attending schools in more rural areas perhaps have less opportunity to visit local retailers at lunchtime, and evidence suggests that teenagers living in rural areas of Scotland eat a diet more in line with recommendations than those living in urban areas (Levin, 2013).

### Scottish case study

The Scottish Government has undertaken significant steps to reform school food. The Schools (Health Promotion and Nutrition) (Scotland) Act 2007 and the Nutritional Requirements for Food and Drink in Schools (Scotland) Regulations 2008 state the energy intake and macro and micro-nutrient content of school meals that should be served over a one-week period. The earlier Hungry for Success (Scottish Government, 2003) report highlighted also the importance of the school environment in encouraging take-up of school meals, an issue revisited in Better Eating, Better Learning (Scottish Government, 2014a).

This current study seeks to examine issues of take-up of FSM, using the Scottish context as a case study. Previous literature has examined why all pupils may or may not choose to eat a meal in the school dining hall; however, there has been little work to investigate why secondary school children who are entitled to receive a free meal take up or do not take up this benefit. This study uses data from the Scottish Healthy Living Survey and School Estates Core Facts Survey, as well as local authority-level information, to examine the following research question: What school and local authority-level characteristics are associated with take-up of FSM in Scottish publically funded secondary schools? Five hypotheses are investigated through the use of multilevel modelling to identify the characteristics most likely to be in pupils’ consciousness as they weigh up the costs and benefits of taking up a FSM.

Hypothesis 1 – FSM take up is more likely where a greater number of pupils are registered for FSM and present in a school, and where a greater proportion of the school roll is registered as eligible to receive FSM. It is perceived that stigma will be less likely in these schools as take up of FSM is more likely to be normalised.

Hypothesis 2 – FSM take-up is more likely when take-up of school meals amongst non-registered pupils is higher. High take-up amongst pupils who have to pay is a likely indicator of popular and high-quality meals. It is also less likely that pupils will need to leave the dining hall to spend time with friends.

Hypothesis 3 – FSM take-up will be higher in schools where the cost of a meal is higher. The higher price suggests that meals are perceived to be of a better quality, and that the benefit received by pupils eligible for FSM is of a higher monetary value.

Hypothesis 4 – FSM take-up is more likely where schools have more modern and appropriate facilities (defined by school condition, suitability and capacity). Pupils will be more likely to want to spend time in these dining hall spaces and more space will be available for pupils to sit with their chosen friends.

Hypothesis 5 – FSM take-up is more likely in schools in more rural local authorities. Pupils in rural areas will have less opportunity to find alternatives to a school meal, and are more likely to be familiar with the healthier foods on offer.

## Method

### Data sources

Data from official Scottish Government sources were used to investigate the hypotheses outlined above. School meals data were obtained from the Scottish Government’s Healthy Living Survey, an annual census of all publicly funded Scottish schools, collected in a single week in February 2014. The data analysed were collected for 342 schools (with an additional 20 being lost to missing data) and 36,086 pupils. Data on school buildings were obtained via the 2014 School Estates Core Facts Survey in which local authorities collect information on the size, condition, suitability and capacity of all publicly funded schools. The survey allows for the ongoing monitoring of school refurbishment. The two datasets were linked using school and local authority names as identifiers.

### Variables

There were two outcome variables based on the take-up of FSM. The continuous outcome was the number of pupils registered for FSM and the number of those present who took an FSM on the census day measured at the school level (Model 1); this variable was skewed and so the log transformation was used to normalise it. It is referred to as Ln(take-up). The dichotomous outcome assigned each individual pupil to yes/no for registered for FSM and present who took an FSM on census day. It is referred to as take-up FSM (Model 2).

Three explanatory variables related to the number of children on the school roll and registered or not registered for FSM. These were the number of children registered for FSM and present on the census day Ln(Registered & Present); the proportion of the school roll registered for FSM Ln(Registered/Roll); and the proportion of the school roll not registered for FSM but taking up a school meal Ln(Take-up Not-Registered).

School refurbishment was measured by examining condition, suitability and capacity. Condition can be defined as the current refurbished state of the school building and suitability defined as whether the school facilities are suitable for its pupils. Both are rated on a 4-point scale (see Table 1 for a further explanation of the ratings). For both condition and suitability, few schools were in category D, so this was combined with C for the empirical analysis. Capacity is the number of children enrolled within the school as a proportion of the school’s official capacity.

**Table 1.** Explanation of condition and suitability ratings.

Rating	Condition	Suitability
A: Good	Performing well and operating efficiently.	Performing well and operating efficiently.
B: Satisfactory	Performing adequately but showing minor deterioration.	Performing adequately but with minor problems.
C: Poor	Showing major defects and/or not operating adequately.	Showing major problems and/or not operating optimally.
D: Bad	Economic life expired and/or risk of failure.	Does not support the delivery of services to children and communities.

An additional explanatory variable was the price paid for a meal. These data were derived by accessing pricing information via local authority or school websites. For many local authorities, meal choices are the same across the area, and a set price is given for the food available. In local authorities where children paid individually for each item bought, a price was calculated from menu price lists based on the price of a main hot meal, a drink and a dessert (fruit or yogurt depending on availability listed – yogurt was always chosen in the first instance). Whilst acknowledging that not all children will select a more expensive hot meal, the price reflects the relative affordability differences across local authorities.

Official Scottish Government data (Scottish Government, 2014b) provided information on the percentage of the population within each local authority falling into one of six categories: Large Urban, Other Urban, accessible Small Towns, Remote Small Towns, Accessible Rural and Remote Rural. We combined ‘neighbouring’ pairs to reduce this to three categories: Urban, Small Town and Rural.

### Analysis

The research design is composed of 36,068 pupils from 342 schools clustered within each of the 32 local authorities in Scotland. Multilevel modelling was used to ensure the estimates and standard errors from the models accounted for this clustering (Leyland & Goldstein, 2001). Model 1 had two levels in the hierarchy – school and local authority. The unit of analysis was school, and weights were used to account for different sizes of the schools. Linear regression with the outcome Ln(take-up) was used. Model 2 had three levels in the hierarchy – pupil, school and local authority. The unit of analysis was pupil. Logistic regression with the outcome take-up FSM was used. Both models adjusted for the school composition variables: the number of children registered for FSM and present on the census day, the proportion of the school roll registered for FSM, the proportion of the school roll not registered for FSM but taking up a school meal, condition and suitability; and local authority contextual variables: cost of a school meal, and proportion of urban/rural.

To deal with potential multiplicative effects and heteroscedasticity, the natural log transformation was applied to original numerical school characteristics for the first three school composition variables. Key assumptions are that under Model 1 Ln(take-up) is assumed, apart from random variation, to be linear in our input variables, whilst, for Model 2, this assumption is made of the log-odds of take-up.

The interpretation of the results from a multilevel model is the same as that for multiple regressions. The parameter estimates from Model 1 are interpreted as the change in the outcome for a unit change in the explanatory variable. The log-odds from Model 2 are interpreted as the change in log-odds of the outcome for a unit change in the explanatory variable. Within the results, odds-ratios are presented and are calculated as exp(parameter estimates) from Table 2.

**Table 2.** Summary statistics, parameter estimates and log-odds for the take-up of FSM adjusting for school and local authority characteristics, 342 secondary schools, Scotland, school year 2013–2014.

Variable	Mean (standard error)	Model 1 Parameter estimates (95% confidence interval) Log(take-up)	Model 2 Log-odds (95% confidence interval) take-up FSM
**School characteristics**			
Ln(Registered & Present) (*n* = 345)	104.83 (3.67)	0.900 (0.822 to 0.978)	−0.556 (−.0866 to −0.246)
Ln(Registered/Roll) (*n* = 348)	15.96% (0.53)	0.102 (0.020 to 0.184)	0.583 (0.211 to 0.955)
Ln(Take-up Not-Registered) (*n* = 344)	40.90% (1.23)	0.071 (0.020 to 0.122)	0.226 (0.030 to 0.422)
Capacity (*n* = 362)	76.18% (0.96)	0.002 (0.000 to 0.004)	0.009 (−0.001 to 0.019)
Condition (*n* = 362)			
A	163 (45%)	−0.086 (−0.206 to 0.034)	−0.007 (−0.440 to 0.426)
B	138 (38%)	−0.055 (−0.120 to 0.010)	−0.091 (−0.434 to 0.252)
C + D	61 (17%)		
Suitability (*n* = 362)			
A	143 (39%)	0.065 (−0.043 to 0.173)	0.138 (−0.270 to 0.546)
B	144 (40%)	0.019 (−0.030 to 0.068)	0.102 (−0.200 to 0.404)
C + D	75 (21%)		
**Local authority characteristics**			
Meal cost (*n* = 362)	£2.08 (0.02)	0.199 (0.015 to 0.383)	0.996 (0.190 to 1.802)
Urban per cent (*n* = 362)	62.20 (1.60)	0.00 (−0.006 to 0.006)	−0.001 (−0.030 to 0.028)
Rural per cent (*n* = 362)	22.97 (1.13)	0.002 (−0.008 to 0.012)	0.003 (−0.038 to 0.044)
**Random effects**			
**Local authority level**			
Variance (SE)		0.021 (0.007)	0.354 (0.118)
ICC		0.292	0.080
**School level**			
Variance (SE)		0.051 (0.001)	0.775 (0.071)
ICC		0.708	0.175
**Pupil level**			
^a^Variance (SE)			π^2^/3 = 3.289
ICC			0.746

^a^For multilevel logistic regression, the variance partition coefficient was calculated by assuming a threshold model and approximating the level 1 (pupil) variance by π^2^/3 = 3.29 as per Snijders and Bosker (1999).

The intraclass correlation coefficient (ICC) measures the extent to which values of the outcome variable are similar for schools clustered within the same local authority. It essentially partitions the unexplained variance into the different levels. It accounts for the clustering by comparing the variance within local authorities with the variance between local authorities. A small value for the ICC implies that the within-local authority variance is much greater than the between-local authority variance.

The ICC for the school level was calculated as Variance(school level)/[Var(school level) + Var(local authority level)]; the ICC for the local authority level was calculated in a similar way.

## Results

Mean take-up levels of FSM were 79% (SD 18.6). Results are structured under each of the hypotheses outlined in the “Introduction” section.

Hypothesis 1: FSM take-up is more likely where a greater number of pupils are registered for FSM and present in a school, and where a greater proportion of the school roll is registered as eligible to receive FSM.

Results show that the Log of Numbers Registered and Present is significantly positive for Model 1. This suggests that the percentage of children taking FSM in schools increases with the numbers registered and present, but not in proportion: thus schools with 10% more pupils registered for FSMs have only around 9% more pupils taking FSMs. Model 2 suggests that the odds of a registered pupil taking an FSM falls when the number of those registered for an FSM and present increases.

The log of the proportion of the school roll that is registered for FSM has a significant and positive coefficient, as does the corresponding odds ratio for Model 2. The positive signs support hypothesis 1in that, under Model 1, Ln(take-up) increases by 0.13 for a unit increase in this variable, whilst, correspondingly, the odds ratio of take-up, increase by 1.5.

Hypothesis 2: FSM take-up is more likely when take-up of school meals amongst non-registered pupils is higher.

In support of hypothesis 2, the coefficient for take-up by non-registered pupils is positive for Model 1. Results suggest that the greater the take-up of school meals amongst pupils not registered for FSM, the more likely are pupils registered for FSM to take-up a meal.

Hypothesis 3: FSM take-up will be higher in schools where the cost of a meal is higher.

In line with hypothesis 3, FSM take-up is higher where the price of school meals is higher for those not registered for FSM, in that, under Model 1, Ln(take-up) increases by 0.22 for a unit increase in this variable, whilst, correspondingly, the log-odds of take-up, increase by 2.0.

Hypothesis 4: FSM take-up is more likely where schools have more modern and appropriate facilities (defined by school condition, suitability and capacity).

For Model 1, results suggest that neither condition nor suitability impacted on FSM take-up. In Model 2, results indicated that FSM take-up was less likely where school condition was highly rated. FSM take-up was lower in schools rated less suitable for their intake of pupils.

Overcrowding was measured by capacity data. Results suggest that capacity had only a small impact on FSM take-up. Model 2 suggests a small positive relationship, with higher school capacity likely to increase a pupil’s likelihood of taking-up an FSM.

Hypothesis 5: FSM take-up is more likely in schools in more rural local authorities.

Results suggest that local authority make-up in terms of urban and rural location had no impact on FSM take-up.

An examination of the share of the variance at each level of the multilevel model revealed that for Model 1, 23.9% of the unexplained variation in log(take-up) is due to the local authority and 76.1% is due to the school.

## Discussion

This study sought to examine the characteristics associated with take-up of FSM amongst Scottish secondary school pupils. Results suggested that take-up increased when more pupils in the school as a percentage of the roll were registered for FSM, when take-up of meals was higher amongst pupils paying for their meal, where meal cost was higher, and when school buildings were rated as more suitable for its pupils.

Utility-maximisation theory provided a framework to investigate take-up of benefits. Within this framework, relevant costs and benefits of take-up were examined. Potential costs associated with taking-up FSM included stigma, an unpleasant school environment, and being separated from friends. Potential benefits included eating high-quality meals and eating with friends.

Literature on take-up of targeted benefits has focused on the stigma associated with that take-up. Previous research around FSM was less clear on whether children or their families experienced stigma in taking up this benefit (Sahota et al., 2014). The results from this study suggest that stigma may be an issue for FSM at secondary school level. As the volume of children registered for FSM increased as a proportion of the school roll, take-up of meals was also likely to increase. This indicates a normalisation effect in schools and for pupils. Conclusions about stigma were complicated by the finding that increases in take-up were not proportional to the number of children registered for FSM and present on the day of the census, and this having a negative impact on an individual pupil’s likelihood of take-up. It is possible that this finding relates more to overcrowding in the dining hall, rather than other explanations. An additional complication is that it is not known which schools operate cashless systems, in which, it is hypothesised that pupils are less likely to encounter stigma around FSM. It would be valuable for this information to be incorporated into future Healthy Living Survey data collection.

The condition of school buildings was not as important as predicted, and neither was capacity. There was, however, evidence that a higher suitability rating positively impacts the probability that pupils take-up an FSM. This result possibly reflects the importance of the way in which schools manage the space they have available for their rolls. Unfortunately, this kind of information has never been operationalised within schools to allow further investigation.

The cost of school meals predicted take-up of FSM. Whilst pupils do not have to pay for their meal if they are registered for FSM, and therefore, pricing should not impact their decision to take-up a meal, results suggest that there is an impact. There are two likely explanations for this. Previous research has found that take-up of benefits is higher when the monetary value of those benefits is greater. Additionally, it is possible that higher meal prices reflect higher quality meals being available to pupils. This explanation is potentially complicated by the fact that some local authorities are more likely than others to subsidise school meals to a greater or lesser extent, and therefore, the price paid at the till may not reflect the cost of the ingredients and foods on offer.

The dispersion of the population in urban or rural areas within local authorities did not have an impact on take-up. This supports findings from Bramley, Lancaster and Gordon (2000), where there were few differences between urban and rural take-up of income support and housing benefit across Scottish local authorities once levels of affluence had been controlled for. In fact, local authority-level variation appeared to be less important in explaining differences in FSM take-up levels than school-level variation.

The results reinforced findings from the literature around the importance of being with friends. FSM take-up was more likely where a high percentage of the school roll were registered for FSM and when take-up was high amongst those not registered for FSM. In these situations, pupils were less likely to have to consider the costs and benefits of eating a meal in the dining hall against spending time with friends purchasing their lunch from local retailers. Macdiarmid et al. (2015) framed eating in school as potentially ‘socially risky’ for pupils due to the value that is attached to spending time with friends. In situations where pupils registered for FSM leave school for lunch, they are potentially weighing up the cost of a meal against the benefits that can be gained from rebelling and resisting the institutional nature of the school dining hall as part of a peer group (Burke 2005; Fletcher, Jamal, Fitzgerald-Yau, & Bonell, 2015; Wills, Danesi, et al., 2015).

De Lange and Lehnin (1976) conceptualise non-take-up of benefits as a failure of the political system and Ringeling (1981) criticise non-take-up as the ‘passivity of the administration’. Within these conceptualisations, welfare benefits are defined as a right, and disengagement from this entitlement highlights cracks in the democratic system. Van Oorschot (1991) argues, using this rationale, that means-tested welfare systems must take on the responsibility of non-take-up. In the case of FSM, this is complicated. Whilst eligible recipients retain the right to a welfare benefit to which they are entitled, they also have the right to develop and express their social identity through alternative means of consumption (Fletcher et al., 2014; Willis, 2003). Following Gustafsson’s (2002) call for children and young people’s voices to be heard in the policy debate around school food reform, we must bear in mind that traditional literature surrounding welfare does not readily accommodate the social complexities involved when children, rather than adults, make the final decision about where they eat. The utility-maximisation model for benefits take-up utilised in this study illustrates this further. While it provides a useful framework and literature to outline the relevant issues, nevertheless, it has the potential to be relatively individualistic in focus and not easily transferrable to FSM. It is likely that lunchtime decisions in secondary schools are the result of group decision-making. The costs and benefits weighed-up are unlikely to be the sum of individual group members’ trade-off processes, and instead are likely to take into account group costs and benefits, as well as numerous compromises and negotiations. Sociological work around engagement with the benefits process, including unusual cases such as FSM, needs to be undertaken to complement the existing economic approaches.

### Limitations

The main limitation of the study is the data available to investigate take-up of FSM. Whilst we were able to locate measures providing insight into issues of stigma and school buildings more generally, there was little additional information around important areas, such as the management of lunchtime, cashless payment systems, local retailers and school ethos surrounding meals. Given that the results suggested that the majority of the variance across the main outcome was found at the school rather than at the local authority level, the collection of additional school-level information is vitally important.

## Conclusion

Take-up of FSM can provide low-income families with an important social benefit, as well as potentially improving health through the nutritionally balanced meals provided in school dining halls. Nevertheless, even under universal systems, take-up will never reach 100%. Issues identified within this study were that stigma does appear to have an impact at secondary level, and that the social benefits of being with friends may, for some pupils, play a more important role than the monetary benefit gained by their family. To investigate the issue of stigma further, more work needs to be carried out to determine take-up of FSM under cash and cashless systems. More generally, results would suggest that schools and local authorities should do all they can to encourage both children paying for their meals, and those receiving FSM to eat in the dining hall by managing the environment in a pupil-friendly manner. This includes facilitating opportunities for young people to contribute to consultations about the menus served and social arrangements in place within dining halls.

## Supplementary data

The underlying research materials for this article can be accessed at http://dx.doi.org/10.5525/gla.researchdata.347
